# PM_2.5_ Concentration Estimation Based on Image Processing Schemes and Simple Linear Regression

**DOI:** 10.3390/s20082423

**Published:** 2020-04-24

**Authors:** Jiun-Jian Liaw, Yung-Fa Huang, Cheng-Hsiung Hsieh, Dung-Ching Lin, Chin-Hsiang Luo

**Affiliations:** 1 Department of Information and Communication Engineering, Chaoyang University of Technology, 168, Jifeng E. Rd., Wufeng District, Taichung 413310, Taiwan; jjliaw@cyut.edu.tw (J.-J.L.); yfahuang@cyut.edu.tw (Y.-F.H.); boom88754032@gmail.com (D.-C.L.); 2 Department of Computer Science and Information Engineering, Chaoyang University of Technology, 168, Jifeng E. Rd., Wufeng District, Taichung 413310, Taiwan; 3 Department of Safety, Health and Environmental Engineering, Hungkuang University, 1018, Sec. 6, Taiwan Blvd., Shalu District, Taichung 433304, Taiwan; andyluo@sunrise.hk.edu.tw

**Keywords:** PM_2.5_ concentration estimation, digital image processing, automatic region of interest selection, data exclusion, linear regression

## Abstract

Fine aerosols with a diameter of less than 2.5 microns (PM_2.5_) have a significant negative impact on human health. However, their measurement devices or instruments are usually expensive and complicated operations are required, so a simple and effective way for measuring the PM_2.5_ concentration is needed. To relieve this problem, this paper attempts to provide an easy alternative approach to PM_2.5_ concentration estimation. The proposed approach is based on image processing schemes and a simple linear regression model. It uses images with a high and low PM_2.5_ concentration to obtain the difference between these images. The difference is applied to find the region with the greatest impact. The approach is described in two stages. First, a series of image processing schemes are employed to automatically select the region of interest (RoI) for PM_2.5_ concentration estimation. Through the selected RoI, a single feature is obtained. Second, by employing the single feature, a simple linear regression model is used and applied to PM_2.5_ concentration estimation. The proposed approach is verified by the real-world open data released by Taiwan’s government. The proposed scheme is not expected to replace component analysis using physical or chemical techniques. We have tried to provide a cheaper and easier way to conduct PM_2.5_ estimation with an acceptable performance more efficiently. To achieve this, further work will be conducted and is summarized at the end of this paper.

## 1. Introduction

Air pollution has been reported to significantly affect human health [1], causing issues such as premature death, bronchitis, asthma, cardiovascular disease, and lung cancer [2]. Pollutants in the air include CO, NO_2_, and particulate matter. Among them, particulate matter with a diameter of less than 2.5 microns (PM_2.5_) is a key component which severely affects human health in many ways. For example, PM_2.5_ aerosols are able to directly enter the lungs through the respiratory tract and affect a person’s health [3]. According to the World Health Organization report, more than 90% of the world’s population inhales large amounts of pollutants every day, which results in approximately seven million deaths each year [4]. Consequently, PM_2.5_ concentration estimation is required and has become an important concern for human health [5,6]. 

Many techniques have been developed to measure the PM_2.5_ concentration, such as the filter-based gravimetric method [7], tapered element oscillating microbalance method [8], beta attenuation monitoring method [9], optical analysis method [10,11], and black smoke measurement [12]. These methods require expensive instruments and professional operations. Some more comprehensive methods analyze the relationship between human activities and PM_2.5_ by satellite and big data [13,14]. However, satellite and big data are not available to the common user. Therefore, a simple and effective method should be sought for PM_2.5_ concentration estimation.

In urban environments, researchers have developed low-cost sensors. These sensors are widely deployed throughout the city to monitor the PM_2.5_ concentration [15]. Although one sensor is low in cost, it is not effective when widely deployed in a city requiring many sensors. The portable PM_2.5_ sensor can be used to monitor the PM_2.5_ concentration at different locations [16]. The portable device reduces the cost of employing a large number of sensors, but requires more manpower to move the sensors. Optical sensors, such as TEOM 1400a analyzer, SDS011 (Nova Fitness, Jinan, China), ZH03A (Winsen, Zhengzhou, China), PMS7003 (Plantower, Beijing China), and OPC-N2 (Alphasense, Braintree, UK), have been introduced to monitor PM_2.5_ [17]. However, these optical sensors are more expensive than ordinary cameras. Since a camera is installed on the top floor of environmental monitoring stations in Taiwan, using the camera to estimate PM_2.5_ is a simpler and more effective approach than employing extra devices. 

It should be noted that air pollution is usually characterized by a poor visibility due to light scattering, such as Rayleigh scattering and Mie scattering, caused by the interaction between light and airborne particles [18]. In other words, the visibility is reduced, as a large amount of aerosol pollution scatter the atmospheric light [19], and vice versa. In previous decades, some researchers proposed methods to estimate the visibility through image processing schemes [20,21]. Recently, an expensive digital camera was used to take high-quality photos for visibility estimation [22]. These studies have shown that image processing schemes can be applied to visibility estimation. Furthermore, it was reported that the PM_2.5_ concentration is related to visibility reduction [23]. However, these studies did not develop image processing technologies to estimate the PM_2.5_ concentration. Therefore, it gives us hope that the PM_2.5_ concentration may be estimated through image processing schemes.

The rapid development of computers, algorithms, and artificial intelligence has meant that image processing methods using machine learning have been widely applied. The main advantage of using machine learning is that it requires training and does not require defining too many features. Two types of the training-based algorithms are neural network methods [24] and linear regression schemes [25]. The neural network methods require a very fast and expensive graphics processing unit [26]. By contrast, compared to neural network methods, the estimation of spatial variations by linear regression could be performed by a consumer computer, as economical and predictive performance were both acceptable [27]. Nowadays, high-quality images can be taken by a commercial digital camera. This facilitates PM_2.5_ concentration estimation by image processing schemes. 

In order to understand which features can affect PM_2.5_ concentrations when using image processing methods, previous research has pointed out that the PM_2.5_ concentrations may affect image characteristics, including the distance, hazy model, entropy, contrast, sky color, and solar zenith angle. It was found that the distance is the feature that has the most influence [28]. This is consistent with the definition of visibility, and a previous study has also shown that visibility can be estimated using high-frequency information from an image [22]. The region of interest (RoI) has also been manually selected to estimate PM_2.5_ concentrations [28]. However, the estimation performance might be degraded because of such a manually selected RoI. Besides, the computational cost might not be cheap, since a support vector regression model with several features was involved in the estimation. To solve these problems, this paper presents an approach to PM_2.5_ concentration estimation, where only a single feature is used and simple linear regression is employed as an estimator. The main contribution of the proposed approach is to use a series of image processing schemes in PM_2.5_ concentration estimation where the images are taken by a consumer camera. It provides a valuable alternative to estimating PM_2.5_ concentration. The main aims of this image-based approach are as follows: (i) to automatically locate the RoI to replace the manual selection of Liu’s work [28]; (ii) to use a single feature for linear regression instead of multiple features in PM_2.5_ concentration estimation, with an acceptable performance; and (iii) to provide a cheaper alternative method with a camera for estimating the PM_2.5_ concentration. This paper is organized as follows. The proposed approach is described in Section 2. In Section 3, real-world data is given to verify the proposed approach. Finally, a conclusion is made in Section 4.

## 2. The Proposed Approach

**Figure 1:**
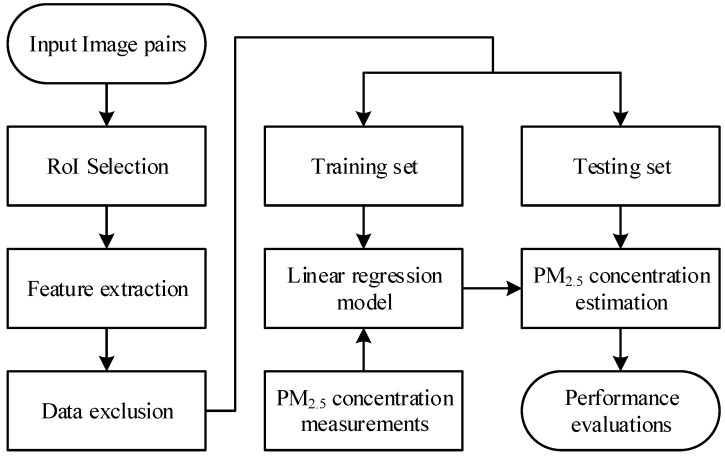
A block diagram of the proposed approach.

There are two stages involved in the proposed approach. In the first stage, a series of image processing schemes are employed to automatically locate the region of interest (RoI) to extract a single feature, which is required in the following stage for PM_2.5_ concentration estimation. In the second stage, a simple linear regression model is used with the training data, which contains pairs of the single feature obtained through the selected RoI and the actual PM_2.5_ concentration measurement. The simple linear regression model is then used in PM_2.5_ concentration estimation with the testing data. The estimated PM_2.5_ concentration is compared with the actual value and evaluated by performance indices. An overall block diagram for the proposed approach is depicted in Figure 1. The details of the proposed approach are described in the following sections. The proposed automatic RoI selection approach is described in Section 2.1, the simple linear regression model is given in Section 2.2, and three performance indices employed to assess the proposed approach are given in Section 2.3.

### 2.1. Automatic RoI Selection

**Figure 2:**
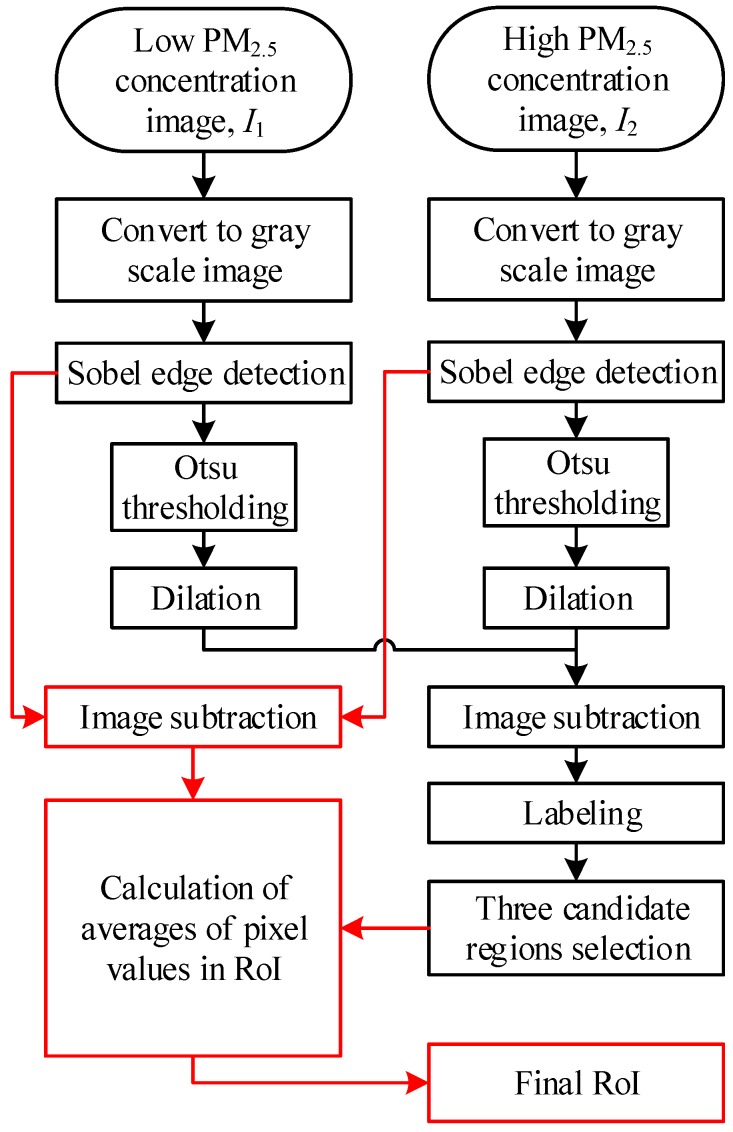
A flowchart of the proposed automatic region of interest (RoI) selection.

**Figure 3:**
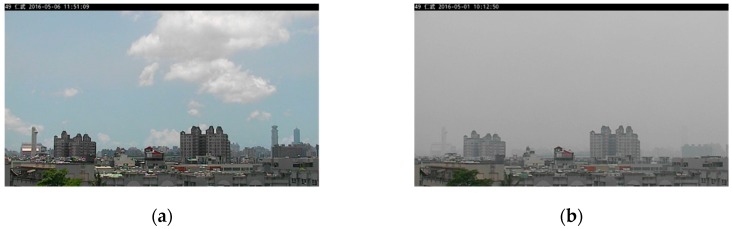
A sample image pair. (**a**) ***I***_1_ (low PM_2.5_ concentration, $1\;\mu g/m^{3}$); (**b**) ***I***_2_ (high PM_2.5_ concentration, $75\;\mu g/m^{3}$).

It should be noted that not all parts of an image are strongly related to the PM_2.5_ concentration. Therefore, selecting an appropriate RoI to estimate the PM_2.5_ concentration is an important step for the successful application of the proposed approach. It is known that some details in the image will be blurred when the PM_2.5_ concentration is high, compared to when there is a low PM_2.5_ concentration. In other words, the pixel value of the images with a high and low PM_2.5_ concentration is different. This also illustrates that not every feature has a good correlation with the PM_2.5_ concentration. It motivates us to use the differences in image pairs of low and high PM_2.5_ concentrations in automatic RoI selection. A flowchart of the proposed automatic RoI selection is depicted in Figure 2. A pair of images, shown in Figure 3a,b, are given to demonstrate how the proposed automatic RoI selection works. Given a pair of images of low and high PM_2.5_ concentrations, both images are converted into gray-level images. The image of a low PM_2.5_ concentration is denoted as ***I***_1_ and the one with a high PM_2.5_ concentration is denoted as ***I***_2_. A series of image processing steps to determine the final RoI is described in the following.

#### 2.1.1. Sobel Edge Detection

As the first step, Sobel edge detection is applied to the image pair, *I*_1_ and *I*_2_, to extract the high-frequency components [29]. In Sobel edge detection, the gradients used in this approach for the *x*-axis and *y*-axis, respectively, are denoted as *G_x_* and *G_y_*, and given as

(1) $$G_{x}=\left[\begin{array}{ccc} -1 & 0 & 1\\ -2 & 0 & 2\\ -1 & 0 & 1 \end{array}\right]{\;\mathrm{and}\;G}_{y}=\left[\begin{array}{ccc} -1 & -2 & -1\\ 0 & 0 & 0\\ 1 & 2 & 1 \end{array}\right],$$

where a 3 × 3 mask is employed. The final magnitude *G_xy_* is calculated as

(2) $$G_{xy}=\left|G_{x}\right|+\left|G_{y}\right|.$$

**Figure 4:**
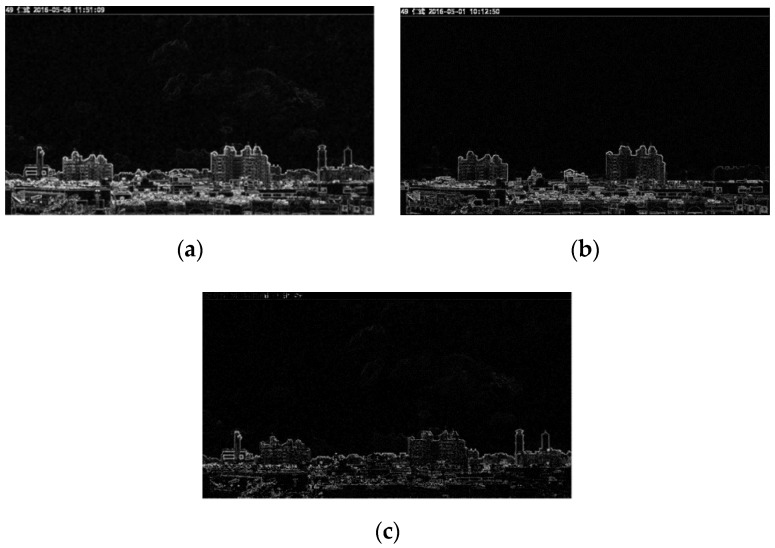
Images after Sobel edge detection. (**a**) ***I***_1__,s_ (low PM_2.5_ concentration); (**b**) ***I***_2__,s_ (high PM_2.5_ concentration); (**c**) the difference of (**a**) and (**b**).

The images produced by Sobel edge detection are denoted as ***I***_1__,s_ and ***I***_2__,s_, and are shown in Figure 4a,b, respectively. In Figure 4, one can see that the low concentration image *I*_1_, after Sobel edge detection, has more details than *I*_2_. This shows that more high-frequency components are contained in *I*_1__,s_ than *I*_2__,s_. The edge detection results of Figure 3a,b are shown in Figure 4a,b, respectively. We can see that two buildings on the right of Figure 3a do not appear in Figure 3b. This is because the PM_2.5_ concentration of Figure 3b is higher than that of Figure 3a. This means that the edges of the two buildings are invisible in Figure 4b. The difference of Figure 4a,b is shown in Figure 4c. The results show that the PM_2.5_ concentration has a significant effect on the high frequency components of images.

#### 2.1.2. Otsu Thresholding

**Figure 5:**
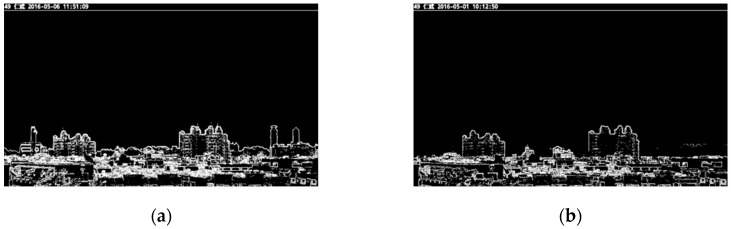
Images after Otsu thresholding. (**a**) ***I***_1__,so_ (low PM_2.5_ concentration); (**b**) ***I***_2__,so_ (high PM_2.5_ concentration).

After Sobel edge detection, Otsu thresholding [30] is applied to the two images in Figure 4 to obtain binary images. In Otsu thresholding, the pixels in an image are separated into two groups based on the histogram. By employing statistical properties, the optimal threshold, where the variance of each group is minimized and the variance between two groups is maximized, is determined. In Otsu thresholding, the weighted sum of the variance between two groups is found as

(3) $$\;\sigma_{w}^{2}{=w}_{0}\left(t\right)\sigma_{0}^{2}\left(t\right){+w}_{1}{(t)\sigma }_{1}^{2}\left(t\right),$$

where $\sigma_{0}^{2}\left(t\right)$ and $\sigma_{1}^{2}\left(t\right)$ represent the variance of each group$,\;{\mathrm{and}\;w}_{0}\left(t\right)$ and $w_{1}\left(t\right)$ are the weights of two groups separated by the threshold *t*, respectively. The weights $w_{0}\left(t\right)$ and $w_{1}\left(t\right)$ are obtained, respectively, as

(4) $$w_{0}\left(t\right)=\overset{t-1}{\underset{i=0}{\sum }}p\left(i\right)$$

and

(5) $$w_{1}\left(t\right)=\sum_{i=t}^{L-1}p(i),$$

where *p*(i) is the probability of the pixel value *i* and *L* is the number of gray levels. The variance between two groups is given as

(6) $$\sigma_{o}^{2}\left(t\right){=\sigma }^{2}-\sigma_{w}^{2}\left(t\right),$$

where $\sigma^{2}$ is the variance of the whole image. Equation (6) can be transformed into

(7) $$\sigma_{o}^{2}\left(t\right){=w}_{0}\left(t\right)w_{1}\left(t\right){\left[\mu_{0}\left(t\right)-\mu_{1}\left(t\right)\right]}^{2},$$

where $\mu_{0}\left(t\right)$ and $\mu_{1}\left(t\right)$ are the means of two groups separated by threshold *t*. The optimal threshold is then found with *t*, which maximizes $\sigma_{o}^{2}\left(t\right)$ in Equation (7). The images ***I***_1__,s_ and ***I***_2__,s_ after Otsu thresholding, are denoted as ***I***_1__,so_ and ***I***_2__,so_ and shown in Figure 5a and Figure 5b, respectively.

#### 2.1.3. Morphological Dilation

Using the obtained binary images, ***I***_1,so_ and ***I***_2,so_, shown in Figure 5, morphological dilation is applied to expand boundaries and to connect neighborhood pixels. The degree of expansion depends on the size of structuring elements. The equation employed for morphological dilation is given below:

(8) $$A⨁{B=\{\mathrm{white}|B}_{x}\cap A\neq \emptyset \},$$

where *A* is the image to be processed and *B* represents the structuring elements.

**Figure 6:**
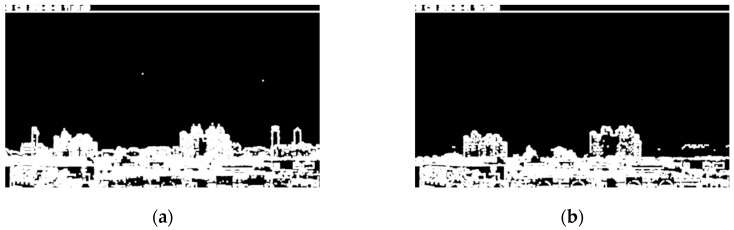
Images after morphological dilation. (**a**) ***I***_1,som_ (low PM_2.5_ concentration); (**b**) ***I***_2,som_ (high PM_2.5_ concentration).

In the proposed RoI scheme, the 3 × 3 mask for structuring elements with all white pixels is used. After morphological dilation, the resulting images are denoted as ***I***_1,som_ and ***I***_2,som_ and shown in Figure 6a and Figure 6b, respectively.

#### 2.1.4. Image Subtraction and Labeling

**Figure 7:**
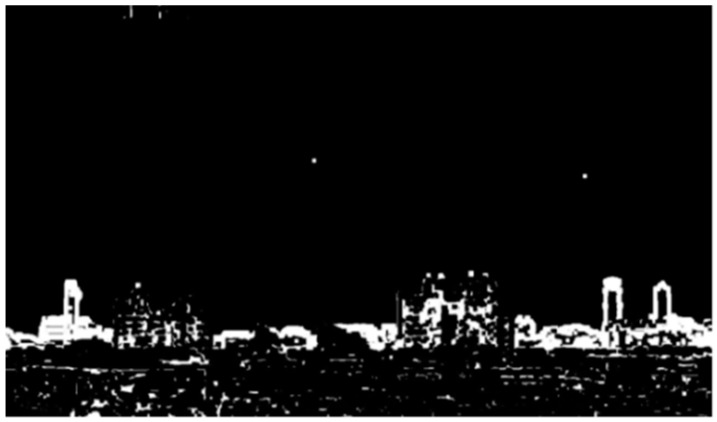
The difference image ***I***_d_ after image subtraction.

**Figure 8:**
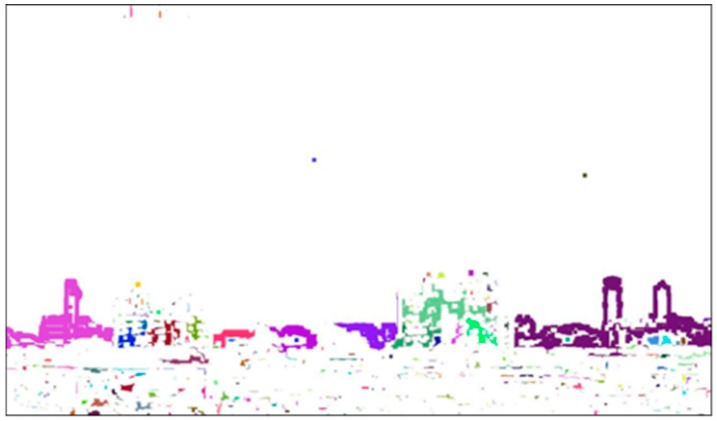
The image ***I***_dl_ after labeling.

In this step, image subtraction is used to obtain the difference image for ***I***_1,som_ and ***I***_2,som_ in Figure 6. Then, a labeling scheme is employed to identify connected pixels. The difference image for ***I***_1,som_ and ***I***_2,som_ is shown in Figure 7, denoted as ***I***_d_, where pixels with the same value in the image pair are eliminated and those with different pixel values remain in a white color. In order to distinguish whether pixels are connected, a labeling scheme [31] is applied to mark the connected pixels by colors. The connected neighborhood pixels are marked with the same color. After labeling, the resulting image, denoted as ***I***_dl_, is as shown in Figure 8. Finally, the labeled regions with the top three largest numbers of pixels are considered as candidate regions of interest.

#### 2.1.5. Selected RoI in the Given Pair of Images

Now, the red flow path shown in Figure 2 will be described. The difference image, denoted as ***I***_sd_, for ***I***_1,s_ and ***I***_2_,_s_ is obtained by image subtraction. Then, the three candidate regions of interest and the difference image ***I***_sd_ are overlapped to select the pixels in the candidate regions of interest. Next, the averages of pixel values in each candidate region of interest are calculated. Then, the RoI with the highest average is determined as the final RoI in the given pair of images, ***I***_1_ and ***I***_2_. This completes the process of automatic RoI selection given in Figure 2 for the given pair of images.

#### 2.1.6. Final RoI Determination

**Figure 9:**
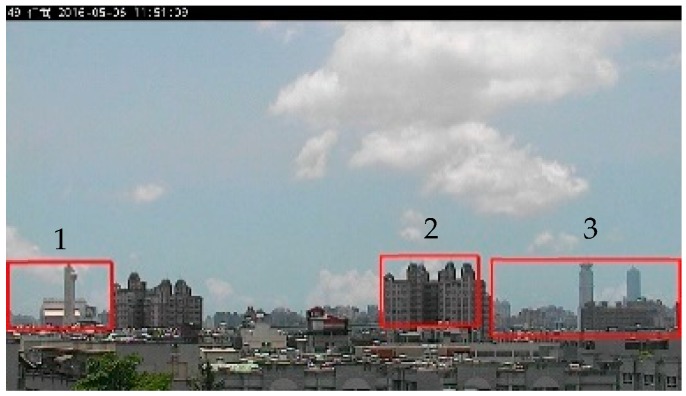
The three candidate regions of interest indicated by red boxes.

**Figure 10:**
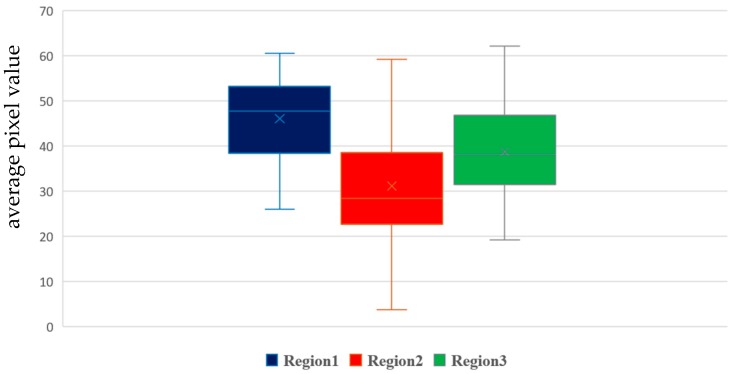
A box plot for three candidate regions of interest.

It needs to be pointed out that the image pair given above is just an example provided to show the process of the proposed automatic RoI selection. In practice, in automatic RoI selection, 30 images with a low PM_2.5_ concentration (≤$5\;\mu g/m^{3}$) and 120 images with a high PM_2.5_ concentration (≥$70\;\mu g/m^{3}$) are randomly selected from the training set. In this study, the images with low and high PM_2.5_ concentrations are paired by combinations. In other words, the 30 × 120 paired images are included in the automatic RoI selection process, as described in Figure 2. By using the averages of 3600 results, the three candidate regions of interest are determined, as shown in Figure 9. The box plot given in Figure 10 shows the range of average pixel values in each candidate RoI. Since Region 1 has the highest average value, it is selected as the final RoI to estimate the PM_2.5_ concentration. The average pixel value within the final RoI will be used as the only single feature for the following simple linear regression model in the proposed approach.

### 2.2. Simple Linear Regression Model

A simple linear regression model, which is a statistical analysis scheme [25], will be used to estimate the PM_2.5_ concentration in the proposed approach. $x_{i}$ is the average pixel value within the final data and $y_{i}$ is the corresponding PM_2.5_ concentration measurement in the training data (where subscript *i* denotes the *i*th sample). It is assumed that these two sequences of data have a linear relation, shown as

(9) $$y_{i}{=\alpha +\beta x}_{i},$$

where α and β are coefficients to be determined. ${ŷ}_{i}$ denotes an estimate of $y_{i}$ (corresponding PM_2.5_ concentration). The estimation error between $y_{i}$ and ${ŷ}_{i}$ is given as

(10) $$\varepsilon_{i}=y_{i}-{ŷ}_{i}.$$

Employing the least squares algorithm to minimize the estimation error, coefficients α and β can be found as

(11) $$\alpha =\sum_{i=1}^{N}y_{i}-\beta \sum_{i=1}^{N}x_{i}$$

and

(12) $$\beta =\frac{\sum_{i=1}^{N}x_{i}y_{i}-\sum_{i=1}^{N}x_{i}\sum_{i=1}^{N}y_{i}}{\sum_{i=1}^{N}x_{i}^{2}-\sum_{i=1}^{N}x_{i}\sum_{i=1}^{N}x_{i}},$$

where *N* is the number of samples. Once the simple linear regression model is obtained, it is employed to estimate the PM_2.5_ concentration with the testing data.

### 2.3. Performance Indices

Inherently, image-based method cannot analyze the ingredients in the air, as in previous works, thus it is hard to define a parameter to show the performance by error. Instead, three overall performance indices are used to evaluate the proposed approach. The first one is the root mean square error (RMSE). It is used to show the error between the recorded value and the estimated value of the proposed method. RMSE is calculated as

(13) $$\mathrm{RMSE}=\sqrt{\frac{1}{N}\sum_{i=1}^{N}{\left(y_{i}-{ŷ}_{i}\right)}^{2}},$$

where $y_{i}$ and ${ŷ}_{i}$ are the true and estimated PM_2.5_ concentrations, respectively. The second performance index is R squared (*R*^2^), which has also been used in previous work [28], and is employed to show the correlation between estimated results and measured values. It is defined as

(14) $$R^{2}=1-\frac{\sum_{i=1}^{N}{\left(y_{i}-{ŷ}_{i}\right)}^{2}}{\sum_{i=1}^{N}{\left(y_{i}-\bar{y}\right)}^{2}},$$

where $\bar{y}$ is the mean of $y_{i}$. *R*^2^ indicates the linearity between ${ŷ}_{i}$ and $y_{i}$. When it is linear, *R*^2^ = 1. The third index is *F*-test, which is the test statistic for an F-distribution under the null hypothesis [32], where the *p*-value indicates the statistical significance; that is, it determines whether the result is beyond chance or not. The *p*-value will be used as an indicator of statistical significance in the following experiments.

## 3. Experimental Results

In this section, the proposed approach is verified by a real-world data set, which is described later in Section 3.1. Then, the results without and with unreliable data exclusion are shown in Section 3.2 and Section 3.3, respectively.

### 3.1. Experimental Data Sets

In the experiments, the images were taken from Renwu Environmental Monitoring Station, Kaohsiung City, Taiwan. A consumer camera was set up at the station and took one image every ten minutes during the period of 7:00 AM to 5:00 PM. In total, 10,084 images were collected from May to October 2016. We did not exclude sampled images of sunny or rainy days. The image data were divided into training and testing data, of which the proportions were 60% and 40%, respectively. The images shown in Figure 3a,b are examples taken from the data set. Furthermore, the hourly PM_2.5_ concentration and relative humidity (RH) in the corresponding area were obtained from the open data released by the Environmental Protection Administration, Executive Yuan, Taiwan [33]. Using the data, a simple linear regression model was obtained and used to estimate the PM_2.5_ concentration by employing the proposed approach.

### 3.2. Results with All Data

**Figure 11:**
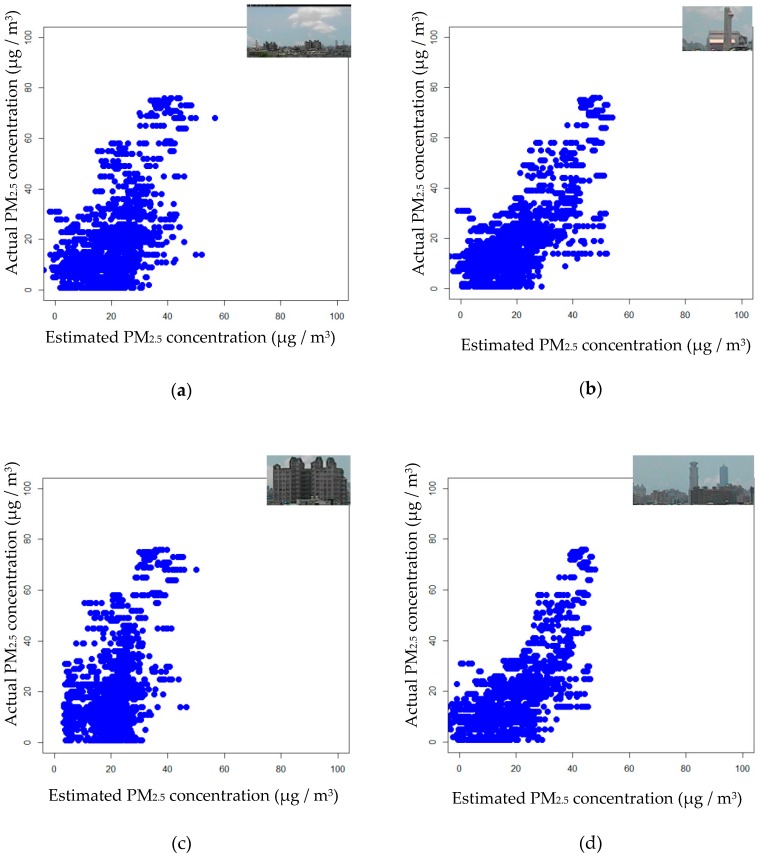
The scatter plots for (**a**) the whole image; (**b**) Region 1 (selected); (**c**) Region 2; (**d**) Region 3.

In this experiment, all of the data set, including 10,084 images, was used. As described in Section 2, three candidate regions of interest were automatically selected and the final RoI was determined by the highest average pixel value among the three candidate regions of interest. Besides, the average pixel value in the final RoI was used as the only single feature. To compare the estimation performances for the whole image, Region 1, Region 2, and Region 3 are presented in Figure 11a–d, which show scattering plots for each case, where the region under consideration is shown in the upper right corner. The three performance indices with all data are displayed in Table 1. Table 1 indicated that Region 1 had a better performance than the other cases. Besides, all results were statistically significant in the F-test. In the case with all data, the highest *R*^2^ = 0.41, which was achieved by Region 1. One may see that the performance of the whole image case is inferior to those for candidate regions of interest. When Regions 1 to 3 are considered, the performance index *R*^2^, from high to low, is Region 1, Region 3, and Region 2. The result is consistent with the priority for the proposed automatic RoI selection. In other words, the proposed automatic RoI selection is appropriate for the given data.

### 3.3. Results with Unreliable Data Exclusion

By conducting experiments, it was observed that two factors may affect the performance of the proposed approach. One is the time difference between the time to take images and the time to measure the PM_2.5_ concentration. For the data set described in Section 3.1, the images were taken every ten minutes, but the PM_2.5_ concentration was collected hourly. In other words, six images were related to only one PM_2.5_ concentration for each hour. When the PM_2.5_ concentration changes within an hour, it might degrade the estimation performance. To solve this problem, the variance of six images taken in the same hour was calculated. When the variance was greater than 1, the images were considered as unreliable data and discarded.

The other factor seen to affect the performance of the proposed approach was the RH. There are many substances, in addition to PM_2.5_, in the atmosphere that affect visibility, such as sulfur oxides, nitrogen oxides, carbon monoxide, and water droplets. It has been observed that PM_2.5_ aerosols are expanded by absorbing water molecules in the air and this affects visibility [34]. It has also been reported that the RH affects PM_2.5_ concentration estimation [28]. Consequently, the effect of RH on PM_2.5_ concentration estimation was considered in the proposed approach.

By conducting experiments, we observed that the estimation performance of the proposed approach was significantly degraded when RH ≥ 65%. Consequently, the data was excluded if its corresponding RH ≥ 65%. Moreover, it should be noted that human health is mostly endangered by a higher PM_2.5_ concentration, instead of a lower one. Consequently, the data with PM_2.5_ concentrations less than 5$\;\mu g/m^{3}$ were excluded. By employing the criteria RH ≥ 65% or PM_2.5_ concentration less than 5$\;\mu g/m^{3}$, 2361 images were excluded from the given data set. With the consideration of data exclusion, the three performance indices were recorded and are presented in Table 2 for all cases, as in Table 1. As seen in Table 2, Region 1 had a better performance than the other cases, as in Table 1. Moreover, all results were statistically significant in the *F*-test. When comparing the results presented in Table 1 and Table 2, one can see that the RMSE and *R*^2^ were obviously improved in all cases with data exclusion. Additionally, Region 1 exhibited the most improvement. The RMSE was reduced from 11.88 to 8.67, while the *R*^2^ increased from 0.41 to 0.73. Again, the results implied that the automatically selected RoI was appropriate in the given example. To sum up, the proposed approach with automatic RoI selection and data exclusion is feasible and has an acceptable performance for PM_2.5_ concentration estimation. By Table 2, one may observe that the performance of the whole image case is inferior to those for candidate regions of interest, as in Table 1. According to the results, the performances from high to low are Region 1, Region 3, and Region 2, which is consistent with the priority for the proposed automatic RoI selection, as shown in Figure 10. Again, the results have verified the feasibility of the proposed automatic RoI selection scheme in the given experiments.

## 4. Conclusions

This paper has presented a simple alternative for estimating the PM_2.5_ concentration in which a series of image processing schemes and simple linear regression are employed. The proposed method uses images with a high and low PM2.5 concentration to obtain the difference between these images. The difference is used to find the RoI. Two main stages are involved in this approach. The first stage includes a series of image processing schemes, which are used to automatically select the final RoI, from which only a single feature is extracted and used in a simple linear regression model. The second stage is employed to find a simple linear regression model with the single feature, by applying the final RoI identified in the first stage. Then, PM_2.5_ concentration estimation is performed. Using an image data set and an open PM_2.5_ concentration data set, experiments were conducted to verify the proposed approach. The results indicated that the proposed approach with the automatically selected RoI achieved the best performance, with *R*^2^ = 0.73. Although the proposed method is not as direct as chemical schemes used to analyze the composition of air, the aim of this paper has been fulfilled, i.e., to provide a simple alternative approach for PM_2.5_ concentration estimation with an acceptable performance. The proposed approach is not expected to replace component analysis using physical or chemical techniques. However, we hope that the proposed method can provide a cheaper and easier way to conduct PM_2.5_ estimation with an acceptable performance more efficiently. To achieve this, further work will be conducted and can be summarized as follows:

1. Since the proposed method uses a fixed camera to capture images at the same location, the influence of images taken in different locations on the results of this study need to be investigated further;
2. Though we have shown that the performance for each candidate RoI is better than the whole image case, it is still worthy to seek a better way to find the final RoI for the performance improvement;
3. In this study, sunny or rainy days are not considered and they will be researched in the future. Besides, other weather factors, such as solar conditions, will be considered in the PM_2.5_ concentration estimation from a higher dimension aspect.

## Figures and Tables

**Table 1:** The performance indices (with all data).

	$\mathrm{RMSE}\;(\mu g/m^{3})$	*R* ^2^	*F*-test
Whole image	14.54	0.11	*p* < 0.0001
Region 1	11.88	0.41	*p* < 0.0001
Region 2	13.53	0.23	*p* < 0.0001
Region 3	12.55	0.34	*p* < 0.0001

**Table 2:** The performance indices (with unreliable data exclusion).

	$\mathrm{RMSE}(\mu g/m^{3})$	*R* ^2^	*F*-test
Whole image	13.17	0.22	*p* < 0.0001
Region 1	8.67	0.73	*p* < 0.0001
Region 2	11.51	0.34	*p* < 0.0001
Region 3	10.76	0.65	*p* < 0.0001
